# Ecological Security Pattern Construction Integrating “Connectivity‐Importance‐Niche” Approach Under Vertical Zonation: A Case Study in Funiu Mountain Area, China

**DOI:** 10.1002/ece3.72848

**Published:** 2026-01-04

**Authors:** Zechen Wang, Jingeng Huo, Zhenqin Shi, Wenbo Zhu, Jiayuan Mao, Sicheng Yan, Shiliang Liu

**Affiliations:** ^1^ Faculty of Geographical Science and Engineering Henan University Zhengzhou China; ^2^ State Key Laboratory of Regional Environment and Sustainability, School of Environment Beijing Normal University Beijing China; ^3^ College of Architecture and Urban Planning Chongqing University Chongqing China; ^4^ Key Laboratory of Geospatial Technology for the Middle and Lower Yellow River Region Henan University, Ministry of Education Kaifeng China; ^5^ Research Center of Regional Development and Planning Henan University Kaifeng China

**Keywords:** comparative analysis, ecological network, ecological niche, ecological security pattern, ecological sources, Funiu Mountain area, vertical zonation

## Abstract

With habitat fragmentation causing the isolation of species populations, the construction of ecological security pattern (ESP) has become a vital approach to safeguarding biodiversity. While identifying ecological sources is considered a key step in ESP construction, existing studies often lack an integrated assessment of ecological sources and overlook the influence of vertical zonation on mountainous ESP. Therefore, this study aims to construct three types of ecological security patterns in the Funiu Mountain region: one based on connectivity, one based on ecological importance, and a third that integrates these with niche suitability, in order to examine how vertical zonation influences their spatial configuration and network performance. The results showed that the spatial distribution of ecological sources, corridors and nodes in ESP_3_ was superior to that in ESP_1_ and ESP_2_. ESP_3_ contained 13 ecological sources (5932 km^2^), 53 corridors (3308 km) and 48 nodes. The area of ecological sources and the length of ecological corridors in EPS_3_ were distributed in unimodal peaks at 800–1200 m, with optimal network connectivity (*α* = 0.6977, *β* = 2.2083, *γ* = 0.8030) and a slight improvement in cost ratio (0.9838). The findings provided a scientific basis for enhancing the conservation and sustainable development of mountain ecosystems from a vertical zonation perspective.

## Graphical abstract

ESPs constructed by different methods showed distinct advantages and limitations. Ecological sources identification improved significantly by coupling three models. Integrated niche suitability model to enhance ecological sources identification. Analyzed mountain ESP based on vertical zonation across different elevation gradients.

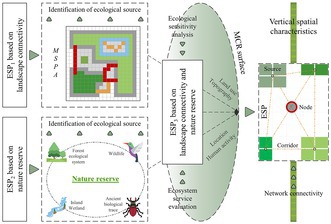

## 1 Introduction

In 2022, the *Kunming‐Montreal Global Biodiversity Framework* adopted at the 16th Conference of parties to the *Convention on Biological Diversity* established a strategic goal to conserve at least 30% of the world's terrestrial and marine areas by 2030, with an important focus on addressing protected area fragmentation and advancing restoration of critical ecosystems. In the *Master Plan of Major National Projects for Protection and Restoration of Important Ecosystems* (2021–2035) proposed in 2020, the Chinese government is systematically advancing top‐level design for ecological security strategies, strengthening institutional safeguards and spatial governance, with the commitment to establish a new national development framework anchored in ecological security pattern (ESP). As a spatial planning paradigm for regional ecological security assurance, ESP shows significant potential in optimizing land use configurations, enhancing ecosystem services and promoting ecological civilization development. Currently, the strategic optimization of ESP to strengthen regional ecological resilience has emerged as an important challenge in global sustainable development (Wang et al. 2020).

Studies on ESP in European and American countries have undergone distinct paradigm shifts: biodiversity conservation dominated in the 1990s (Hess and Fischer 2001), evolved into ecosystem service valuation in the 2000s (Lai et al. 2025; Opdam et al. 2006; Wang, Chang, et al. 2025; Wang, Liu, et al. 2025; Wang, Shen, et al. 2025) and expanded to ecological security post‐2010 (Kenchington and Day 2011). In contrast, Chinese studies have concentrated on regional ESP (Ma et al. 2004), urban ecological network planning (Dai et al. 2020) and national land‐space ecological restoration strategies (Ma et al. 2021). Methodologically, the establishment of ESP is divided into ecological sources identification, corridor extraction and node localization (Fu et al. 2020; Ding et al. 2022). The Minimum Cumulative Resistance (MCR) model remains predominant for ecological corridor extraction through cost‐path analysis (Yang, Li, et al. 2023; Yang, Ma, et al. 2023; Chen et al. 2021), while node localization typically involves spatial coupling between MCR surface geomorphological features (e.g., ridge‐valley lines) and corridor/road network topology (Yang, Li, et al. 2023; Yang, Ma, et al. 2023). Notably, ecological sources identification methods are diverse. Early studies mechanically selected large continuous ecological lands (Zhu et al. 2020) or administratively delineated ecological reserves (Vergnes et al. 2013). However, single‐criterion approaches often fail to capture the mismatch between structural connectivity and functional ecological processes, leading to biased identification of key ecological sources in vertically heterogeneous mountain landscapes (Wei et al. 2024). Recent advances emphasize the integration of multidimensional attributes, including ecological importance assessment (Peng et al. 2019), sensitivity analysis (Chen et al. 2022) and connectivity measures (Rahaman et al. 2023). But their systematic integration is still lacking in existing studies.

The comprehensive ecological niche suitability evaluation model is designed to identify areas that provide the most favorable environmental conditions for maintaining species survival, ecological processes and long‐term habitat stability. Such models typically integrate multiple dimensions of ecological information, including biophysical factors, ecological sensitivity metrics and indicators related to ecosystem functioning. Recent European practice has also begun to incorporate ecosystem service information into spatial ecological assessments (Hernandez and Camerin 2024).

In the plain areas, ESP is mainly composed of farmland ecosystem, supplemented by natural patches such as forests and wetlands (Qiao et al. 2024). It is facing the continuous impact of population agglomeration and high‐speed economic development pressures. The urbanization process and the advancement of agricultural production have led to continuous erosion of natural ecological spaces, which significantly decreases the connectivity between ecological sources and threatens biodiversity conservation (Guo et al. 2021; Wang et al. 2021). In the coastal areas, ESP is mainly composed of wetlands, mangroves and seagrass beds with unique ecological functions (Qian et al. 2023). They play important roles in maintaining ecological balance, resisting typhoon disasters and providing carbon sink services (Jagtap et al. 2003; Lai et al. 2025). In recent years, some coastal areas have effectively curbed disorderly expansion of reclamation by implementing ecological protection red lines and ecological restoration green lines, enhancing structural stability and ecological service functions (Wang et al. 2015, 2022). However, in the context of global climate change, rising sea levels pose threats to long‐term survival of coastal areas, and increased frequency of tidal intrusions leads to vegetation degradation and even intensifies the risk of ecosystem extinction (Carrasco et al. 2016). Compared to high development pressure in plain areas and land‐sea interaction effects in coastal areas, ESP of mountain areas shows unique vertical zonation characteristics. Forests, rivers and grasslands formed with mountains as the backbone, maintain hydrological regulation, water conservation and biological habitat functions through complex material and energy cycles (Lv et al. 2022). Vertical terrain and geomorphology endow its ecosystem diversity, but also expose it to frequent geologic disasters and significant differences in human disturbances. As the concept of ecological protection deepens, the construction of ESP in mountain areas needs to pay more attention to coupling relationship between natural and human systems, and to balance resource development and ecological protection (Yang et al. 2025). The distinct topographical features require differentiated management strategies for ESP, and multi‐dimensional comparative study and targeted conservation design are necessary.

Mountain ecosystems are characterized by significant vertical zonation, with their ecological processes and service functions showing distinct differences with altitude and terrain conditions. For example, low‐altitude areas often suffer from high‐intensity human activity interference, while high‐altitude areas face challenges from harsh climatic conditions and increased ecosystem fragility. Therefore, to achieve effective ecological protection, ESP construction must fully account for the impact of vertical gradients on ecosystem structure and function. In this study, Morphological Spatial Pattern Analysis (MSPA), ecological niche model and MCR model were used to identify ecological sources, corridors and nodes in different types of ESPs, and detailed analyses were conducted on their spatial distribution characteristics and ecological function differences at various altitudinal gradients. The results aim to reveal the distribution patterns of ESP in vertical zonation of mountains, providing scientific support for formulating differentiated ecological protection strategies and offering references for the sustainable development in similar mountain areas globally.

## 2 Materials and Methods

### 2.1 Study Area

**FIGURE 1:**
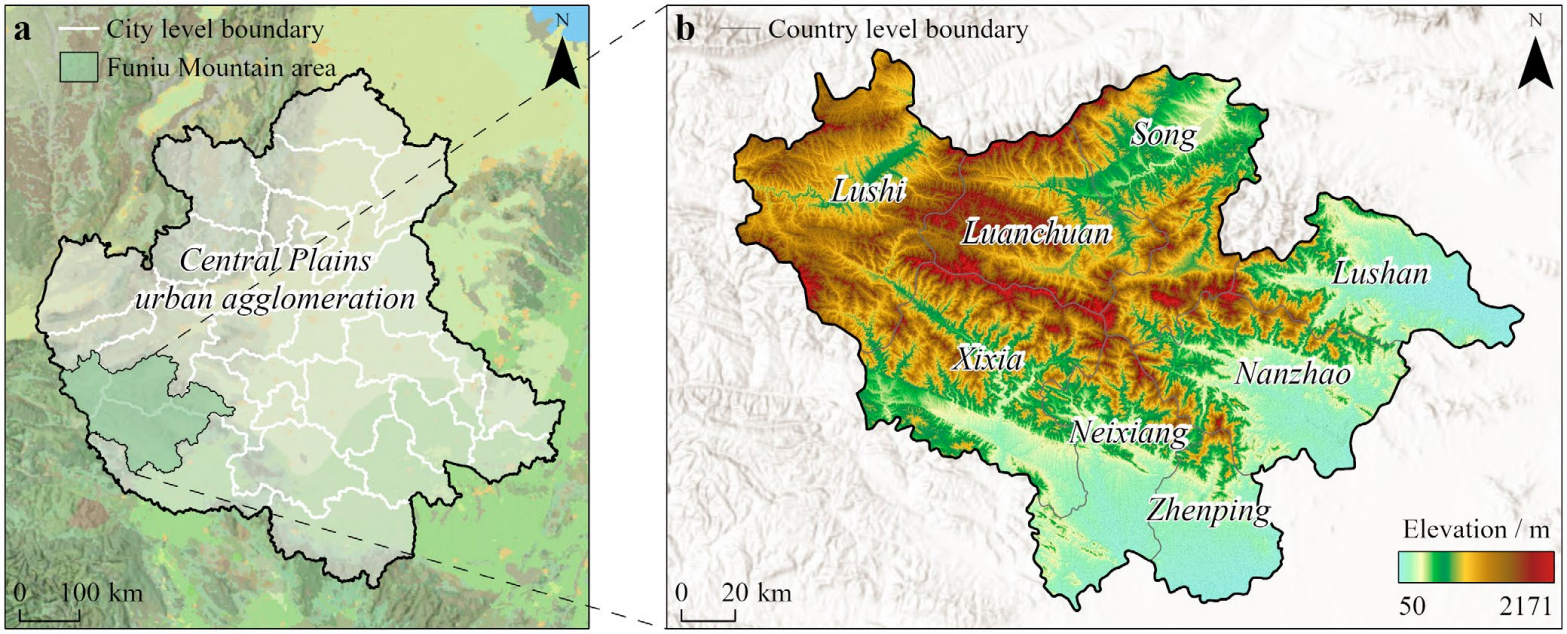
Geographic location of Funiu Mountain area (a, location of Funiu Mountain area in the Central Plains urban agglomeration; b, elevation distribution of counties in Funiu Mountain area).

The Funiu Mountain area (110°30′–113°30′ E, 32°45′–34°20′ N), located in the eastern extension of the Qinling Mountains, covers an area of 21,600 km^2^. It serves as the confluence point where the Yellow River and Huai River meet, as well as where subtropical and warm temperate zones intersect. It is one of the three major ecological barriers for the Central Plains urban agglomeration (Figure 1). The Funiu Mountain area has seven natural reserves and rich contiguous forest resources, but its ecosystem is very fragile (Wang et al. 2023). In the past 30 years, driven by the heterogeneity of the vertical gradient environment, there has been a significant transformation in land use pattern, characterized by continuous shrinkage of ecological spaces, disorderly expansion of urban spaces and increasing intensity of human interferences (Zhang et al. 2022).

In addition to biophysical factors, territorial transformations in the Funiu Mountain region have also been shaped by major national ecological restoration policies. Since the late 1990s, programs such as the *Natural Forest Protection Program* and the “return of farmland to forest” initiative have aimed to restore vegetation cover, reduce soil erosion and alleviate pressure on mountainous ecosystems (Wu et al. 2025). Although these policies have contributed to ecological recovery in certain areas, recent assessments indicate that their long‐term effectiveness remains uneven due to variation in implementation intensity, monitoring continuity and local socioeconomic conditions. At the same time, rapid urbanization, intensive agriculture and expanding infrastructure networks continue to exert strong pressures on ecological space, creating a complex socio‐ecological landscape (Morán Uriel et al. 2024). These interactions illustrate that the Funiu Mountain region reflects broader national dynamics of economic development, demographic change and ecological resilience, thereby reinforcing the necessity of constructing ESP that integrate both natural and human‐driven processes.

### 2.2 Data Sources

The fundamental data are described in Table 1, with the coordinate system of geographic element data standardized to WGS 1984. The land use types of raw data were reclassified into paddy field, dryland, closed forestland, shrub, sparse forestland, grassland, water area, construction land and unused land to align with the objectives of this study. The 30 m spatial resolution adopted in this study is commonly used in regional ecological assessments and provides an appropriate level of detail for identifying ecological sources, landscape patterns, and environmental gradients across the Funiu Mountain region. At the regional scale, this resolution effectively captures major topographic structures, vegetation patterns, and land use transitions, which are the key factors influencing ecological security pattern construction (Córdoba Hernández and Camerin 2023). Although finer‐scale data can offer additional detail in highly heterogeneous mountain environments, the datasets used here are consistent with established practices in large‐area ecological studies and provide a reliable basis for the analyses conducted in this research. Recent European work has also shown that improving spatial detail can further refine ecosystem assessment, which offers useful reference for future studies. Data on invasive species distribution were not available at the spatial resolution required for this study. It was not included in the resistance surface or source identification.

**TABLE 1:** Data sources and description.

Data	Resolution	Year	Source
Nature reserve	—	2020	China Natural Reserve Biological Specimen Resource Sharing Platform (http://www.papc.cn)
Land use	30 m × 30 m	2020	Resource and Environmental Science Data Platform (http://www.resdc.cn)
Digital elevation model	30 m × 30 m	2020	
Normalized difference vegetation index (NDVI)	30 m × 30 m	2005–2020	
Soil texture	30 m × 30 m	2020	
Mean annual temperature	30 m × 30 m	2005–2020	
Mean annual precipitation	30 m × 30 m	2005–2020	
Net primary productivity (NPP)	30 m × 30 m	2005–2020	
Nighttime light index	1 km × 1 km	2020	Geospatial Data Cloud (http://www.gscloud.cn)
Road	—	2020	OpenStreetMap (https://www.osgeo.cn)
Administrative region	—	2020	Colorado School of Mines (https://eogdata.mines.edu)

### 2.3 Methods

**FIGURE 2:**
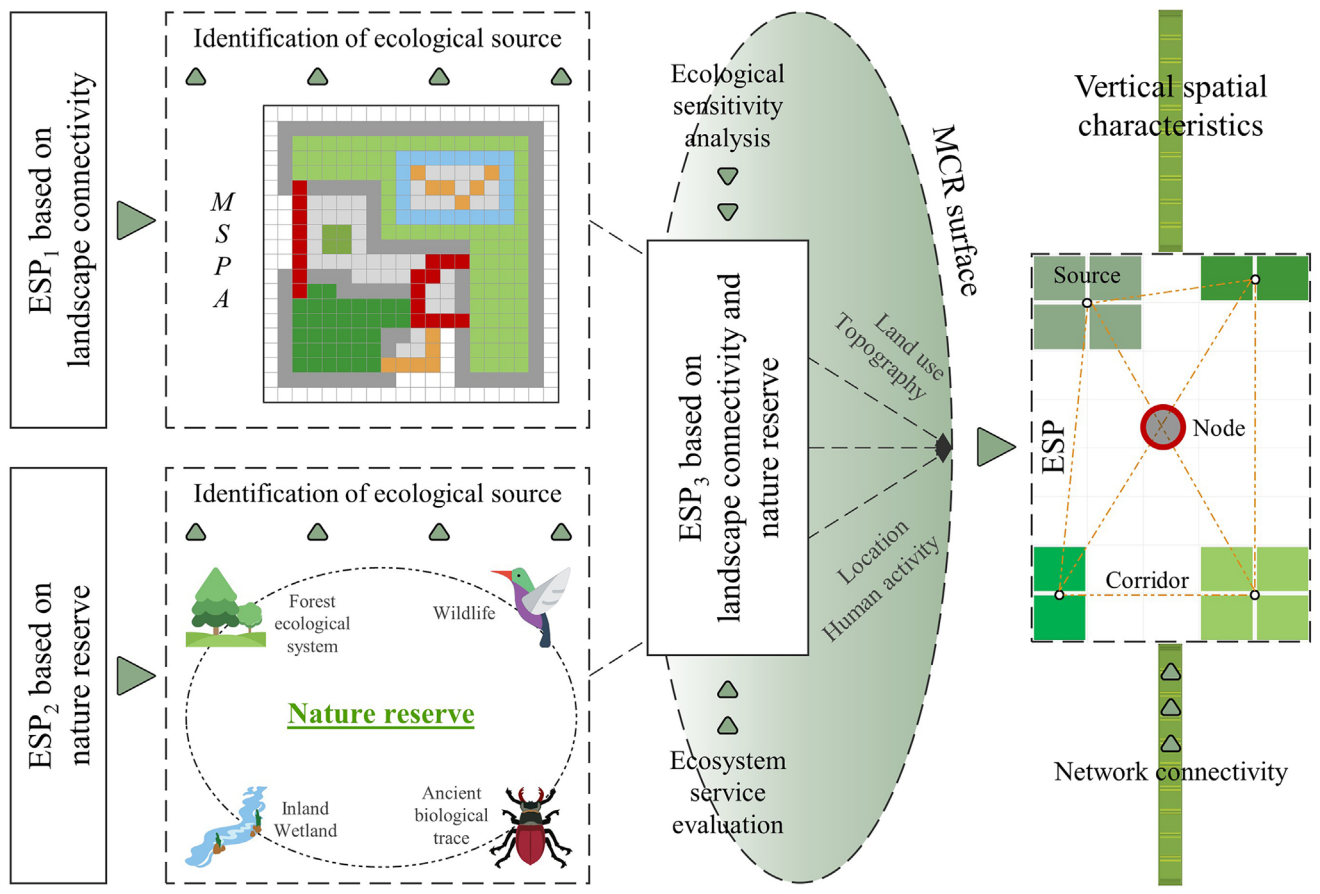
The flowchart for constructing ESP.

Ecological source is the core foundation of ESP construction, and their selection criteria and spatial combination have determinant impacts on ecological functions of ESP (Cao et al. 2024). Differentiated ecological sources identification processes were conducted to construct three typical ESPs in this study: Sources were identified based on connectivity theory (i.e., MSPA) for ESP_1_; Sources were identified based on the scope of legally designated nature reserve for ESP_2_; The selective process of potential ecological sources was optimized by combining both connectivity and nature reserve for ESP_3_. They simultaneously integrated ecosystem service evaluation and ecological sensitivity analysis to identify optimal ecological sources. Land use types, topographic factors (elevation and slope), location distance (road/water proximity) and human activity intensity indicators (nighttime light index) were used to construct MCR surface, quantitatively characterizing spatial heterogeneity resistance of ecological flow diffusion. Minimum cost‐path algorithm and hydrological analysis were used to extract ecological corridors and locate ecological nodes, which together with ecological sources constitute a complete ESP system. Network connectivity of ESP_1_, ESP_2_ and ESP_3_ was evaluated and analyzed for their vertical divergence pattern and spatial distribution characteristics based on a 200 m altitudinal gradient. The study process is shown in Figure 2.

#### 2.3.1 Identification of Ecological Sources

##### 2.3.1.1 Identification of Ecological Sources Based on Connectivity

MSPA can classify binarized raster images based on morphological principles, and thus measure, select and segment patches to distinguish spatial distribution of landscape types with different attributes (Vogt et al. 2007). It was conducted in Guidos Toolbox software (https://forest.jrc.ec.europa.eu). Foreground and Background settings for MSPA are based on previous research (Wang et al. 2023). Foreground connection, edge width, conversion rate and opening were set to 4, 1, 1, and 1, respectively.

##### 2.3.1.2 Identification of Ecological Sources Based on Importance

Natural reserves typically possess intact climax community structures, such as primary forests and wetland complexes (Peng et al. 2019). Within them, processes of material circulation, energy flow and hydrological regulation are maintained and show positive ecological flow output (Yuan et al. 2021). Therefore, natural reserves meet the attributes of ecological sources in ESP. Seven natural reserves were selected as potential ecological sources for ESP_2_, encompassing categories of forest ecological system, inland wetland, wildlife and ancient biological trace. In this process, Xiong'er Mountain and Dinosaur Egg Fossil Group nature reserves, located adjacent to the geographical boundary of Funiu Mountain area, were included in the analysis due to consideration of spatial correlation.

##### 2.3.1.3 Incorporating Niche Suitability Evaluation Into Ecological Sources Identification

The ecological niche model originates from Hutchinson's multidimensional hypervolume theory, which aims to accurately determine the most suitable position of ecological elements (Coro et al. 2013). It was used as a guide to evaluate potential ecological sources for constructing ESP. In this process, ecosystem service and ecological sensitivity were considered to determine optimal ecological sources (Table 2). In ecosystem service dimension, biodiversity maintenance, water supply and soil and water conservation were emphasized (Wong et al. 2015). In ecological sensitivity dimension, soil and water loss sensitivity, land desertification sensitivity and human activity intensity were emphasized (Jin et al. 2021). The spatial overlap degree (comprehensive ecological niche) between actual ecological niche and ideal was calculated, and those with a value greater than 0.4 were judged as optimal ecological sources. The formulas are as follows:

(1) $$F_{k}=\sum_{k=1}^{n}W_{k}\times F_{k}$$

(2) $$F=\left\{\begin{array}{c} 0\;D_{\text{kmin}}>X_{k}\\ \begin{array}{cc} X_{k}/D_{\text{kopt}} & D_{\text{kopt}}>X_{k}\geq D_{\text{kmin}} \end{array}\\ \begin{array}{cc} 1 & X_{k}\geq \end{array}D_{\text{kopt}} \end{array}\right.$$

where *F*_*k*_ is the ecological niche of the *k*‐th indicator; *X*_*k*_ is the actual niche; *D*_*kpot*_ is the ideal niche; *D*_*kmin*_ is the minimum ecological niche; *F* is the comprehensive ecological niche; *W*_*k*_ is the weight.

**TABLE 2:** Ecosystem service evaluation and ecological sensitivity analysis.

Dimension	Indicator (weight)	Formula and processing	Description
Ecosystem service	Biodiversity maintenance (0.25)	$NPP_{\mathrm{mean}}\times F_{\mathrm{pre}}\times F_{\mathrm{tmp}}\times \left(1-F_{\mathrm{ele}}\right)$	*NPP*_*mean*_: Mean annual of *NPP* *F*_pre_: Mean annual precipitation *F*_tmp_: Mean annual temperature *F*_ele_: Elevation *F*_sic_: Soil infiltration capacity factor *F*_slo_: Slope *K*: Soil erodibility index, calculated and coefficient corrected by erosion‐productivity model *R_i_*: Rainfall erosivity factor *K*_*i*_: Soil erodibility factor *LS*_*i*_: Slope length factor *I*_*i*_: Dryness index *W*_*i*_: Number of days with sandstorms *D*_*i*_: Soil texture *C_i_*: Vegetation cover factor *SCLE*: Equivalent area of construction land *S*: Total area of the region
	Water supply (0.45)	$NPP_{\mathrm{mean}}\times F_{\mathrm{sic}}\times F_{\mathrm{pre}}\times \left(1-F_{\mathrm{slo}}\right)$	
	Soil and water conservation (0.30)	$NPP_{\mathrm{mean}}\times \left(1-K\right)\times \left(1-F_{\mathrm{slo}}\right)$	
Ecological sensitivity	Soil and water loss sensitivity (0.26)	$\sqrt[{4}]{R_{i}\times K_{i}\times {\mathrm{LS}}_{i}\times C_{i}}$	
	Land desertification sensitivity (0.55)	$\sqrt[{4}]{I_{i}\times W_{i}\times D_{i}\times C_{i}}$	
	Human activity intensity (0.19)	$\frac{\text{SCLE}}{S}\times 100\%$	

*Note:* The weights are determined by analytic hierarchy process (AHP).

#### 2.3.2 Extract Ecological Corridors and Nodes

MCR model can calculate the minimum resistance to be overcome during their horizontal movement of organisms from source to destination (Li, Huang, Wu, and Yang 2023). It quantifies the cost of diffusion from a certain area to its surroundings and the feasibility of spatial crossing, emphasizing the cumulative effect of resistance in space (Knaapen et al. 1992). MCR model was used to extract ecological corridor for ESP. Resistance indicators, including land use, elevation, slope, road proximity, water proximity and nighttime light index, were classified and assigned values to generate a continuous MCR surface (Table 3). On this basis, potential migration paths between ecological source were calculated by minimum cost‐path algorithm in ArcGIS software (https://www.esri.com). After integration and deduplication, the final ecological corridors were extracted (Li, Huang, and Yang 2023). Furthermore, MCR surface was transformed by hydrological analysis of anti‐topography to extract ridge lines (low resistance channels) and valley lines (high resistance channels) (Guo et al. 2025). The intersections of ecological corridors and ridge lines were located as strategic ecological nodes, which play key hub roles in biological migration and contribute significantly to maintaining regional ecological connectivity. The intersections of road networks and valley lines were located as artificial environmental ecological nodes. Due to intense human activities, these nodes typically face significant pressure for biodiversity conservation and need to be given priority protection.

**TABLE 3:** Construction of MCR surface.

Indicator	Resistance classification and assignment										Weight	References
	10	20	30	40	50	60	70	80	90	100		
MSPA landscape element	Core and bridge	Edge and islet	Branch and loop	—	—	—	—	—	Background	—	0.23	Tao et al. (2022)
Land use/dimensionless	Closed forestland	Sparse forestland and grassland	Water area	Paddy field	Dryland and unused land	—	—	—	Construction land	—	0.25	Huang et al. (2019)
Elevation/m	≤ 373	—	373–666	—	666–959	—	959–1275	—	> 1275	—	0.19	Huo et al. (2023)
Slope/°	≤ 8	8–15	—	15–25	—	25–40	—	> 40	—	—	0.14	
Road proximity/m	—	> 1500	—	1000–1500	—	500–1000	—	≤ 500	—	—	0.07	Liu et al. (2023)
Water proximity/m	—	≤ 100	—	100–400	—	400–800	—	> 800	—	—	0.04	
Nighttime light index/normalization	—	≤ 10	—	10–35	—	35–55	—	> 55	—	—	0.08	Han et al. (2012)

*Note:* The weights are determined by AHP.

#### 2.3.3 Network Connectivity

Based on graph theory, the point‐line combination of ecological nodes and ecological corridors was used to evaluate closure degree (*α*), complexity (*β*), and connectivity degree (*γ*) of ESPs formed by different ecological sources, i.e., network connectivity evaluation (Zhou et al. 2024). The high value of *α* indicates efficient material circulation and energy flow in ecological corridors. The high value of *β* indicates multiple linkage pathways in ecological sources, leading to strong stability of ESP. The high value of *γ* indicates a close relationship between ecological nodes, which is beneficial for migration, diffusion, and genetic exchange of organisms. Additionally, Cost ratio reflects average resistance encountered in activities such as biological migration. The low value of Cost ratio indicates that the cost of constructing and maintaining ESP is low, with high ecological and economic feasibility. The formulas are as follows:

(3) $$\alpha =\frac{L-V+1}{2\times V-5}$$

(4) $$\beta =\frac{L}{V}$$

(5) $$\gamma =\frac{L}{3\times \left(V-2\right)}$$

(6) $$\text{Cost ratio}=1-\frac{L}{C}$$

where *L* and *V* is number of ecological corridor and ecological node, respectively; *C* is the length of ecological corridor.

## 3 Results

This section presents the results in three steps. First, we describe the distribution characteristics of ecological sources and corridors under the three ESP types. Second, we compare the structural connectivity and network properties of ESP_1_, ESP_2_ and ESP_3_. Finally, we analyze how these spatial patterns vary along the vertical zonation of the mountainous landscape. Together, these results provide the basis for understanding the integrated performance of the ESPs.

### 3.1 Differences of Ecological Sources Distribution

**FIGURE 3:**
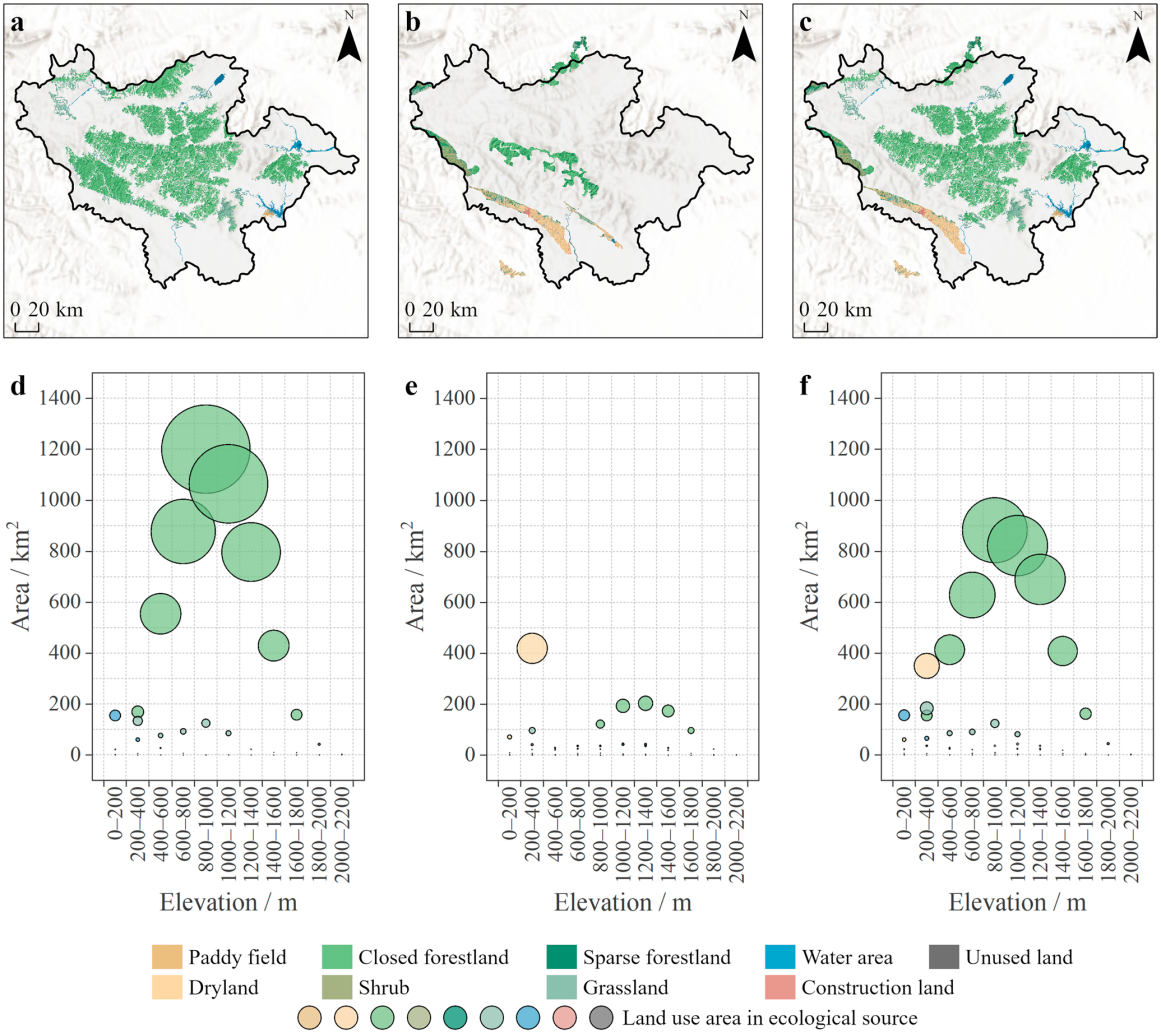
Vertical pattern differences in ecological sources (a–c, land use distribution patterns on ecological sources according to connectivity, nature reserves, and a combination of them complemented by ecosystem service evaluation and ecological sensitivity analysis; d–f, land use areas of 200 m altitudinal gradient in ecological sources).

For ESP_1_, the area of ecological sources identified based on connectivity reached 6149.62 km^2^, with closed forestland as the main land use type and concentrated distribution in the central part (Figure 3). Typical water areas such as Luhun Reservoir in the northeast, Zhaopingtai Reservoir in the east, Yahukou Reservoir in the southeast, and large areas of paddy field adjacent to them were identified as ecological sources due to their transitional role in ecological circulation. Vertical altitudinal gradient analysis showed that the land use type in ecological sources gradually tended to be singular at 0–800 m, and the proportions of closed forestland and grassland significantly increased. They peaked at 800–1000 m and had optimal connectivity.

For ESP_2_, the area of ecological sources identified based on nature reserves reached 6149.62 km^2^, with dryland as the main land use type and concentrated distribution in the southwest. Nature reserves of ancient biological trace and wildlife, such as Dinosaur Egg Fossil Group, were mainly distributed at 0–400 m. Nature reserves of forest ecological systems, such as Baotianman and Xiong'er Mountain, were mainly distributed at 800–1800 m.

For ESP_3_, the area of ecological sources was adjusted to 5928.66 km^2^ by optimization from ecosystem service evaluation and ecological sensitivity analysis. It mainly included dryland (7.67%), closed forestland (71.04%), grassland (10.39%) and water area (4.34%). Dryland and water area can maintain agricultural landscape and regulate local microclimates at 0–1000 m. The composite system of closed forestland and grassland can promote carbon sequestration enhancement, soil and water conservation, and other ecological regulatory services at 1000–1800 m. However, the simplicity of ecosystem composition and harsh natural environment at 1800–2200 m limit ecological service function and connectivity, making it challenging to form complete and efficient ecological sources.

### 3.2 Differences of ESP Distribution

**FIGURE 4:**
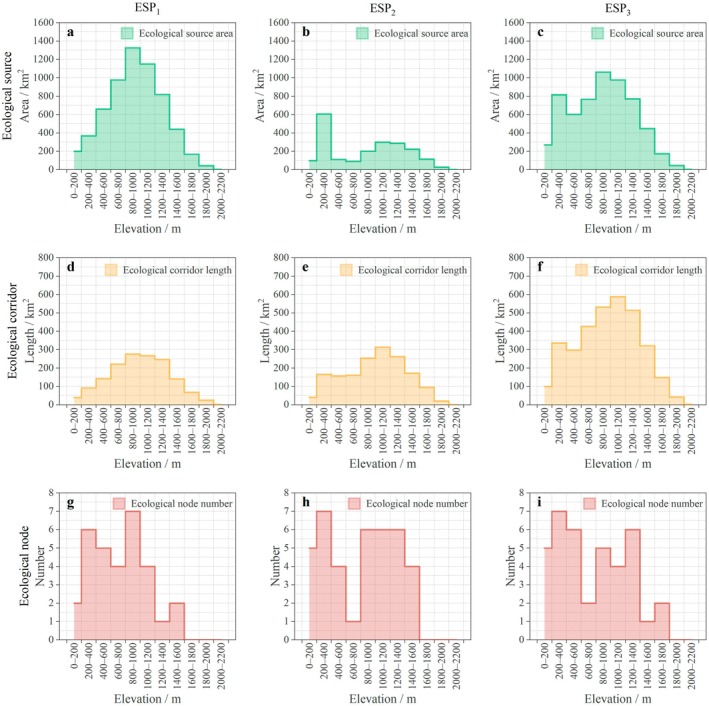
Vertical pattern differences in ESP (a–c, the area of ecological sources in ESP_1_, ESP_2_ and ESP_3_ at different altitudinal gradients; d–f, the length of ecological corridors in ESP_1_, ESP_2_ and ESP_3_ at different altitudinal gradients; g–i, the number of ecological nodes in ESP_1_, ESP_2_ and ESP_3_ at different altitudinal gradients).

In ESP_1_, the area of ecological sources and the length of ecological corridors showed significant unimodal distributions with increasing altitude, peaking at 800–1000 m (1325.87 km^2^ and 276.02 km, respectively) (Figure 4). The microclimate conditions of mean annual temperature of 12.58°C and mean annual precipitation of 722.52 mm within this altitude range effectively promote the stable succession of vegetation communities, as evidenced by the significantly higher mean NDVI (0.89) and mean NPP (669.52 g C/m^2^) than those in other altitude ranges. Meanwhile, the nighttime light index was only 0.04 nW/cm^2^/sr at 800–1000 m, which was significantly lower than that of 0.89 nW/cm^2^/sr at 0–200 m. Less human activity was favorable to maintain the integrity of landscape structure.

In ESP_2_, the area of ecological sources and the length of ecological corridors peaked at 200–400 and 1000–1200 m, respectively. However, the maximum source area was decreased by 719.68 km^2^ and the longest corridor was shortened by 23.57 km. The spatial connectivity of ESP_2_ was poor, and the explanatory power of altitude on its spatial differentiation was weak.

The area of ecological sources and the length of ecological corridors in ESP_3_ showed unimodal distributions similar to that of ESP_1_, peaking at 800–1000 m (1062.05km^2^) and 1000–1200 m (587.79 km), respectively. A composite ecological network conforming to the characteristics of mountain vertical zonation was formed within this altitude range (mean slope is 21.52° and mean aspect is 176.74°). There was a significant nonlinear relationship between ecological nodes and altitude, with nodes in ESP_1_ mainly concentrated at 800–1200 m (accounting for 22.58%), while nodes in ESP_2_ and ESP_3_ were mainly concentrated at 200–400 m (accounting for 17.95% and 18.42%, respectively). The interaction between topography and human activities may jointly have complex impacts on the spatial positioning of ecological nodes, and it is not solely controlled by the altitudinal gradient.

### 3.3 Network Connectivity in ESP

**FIGURE 5:**
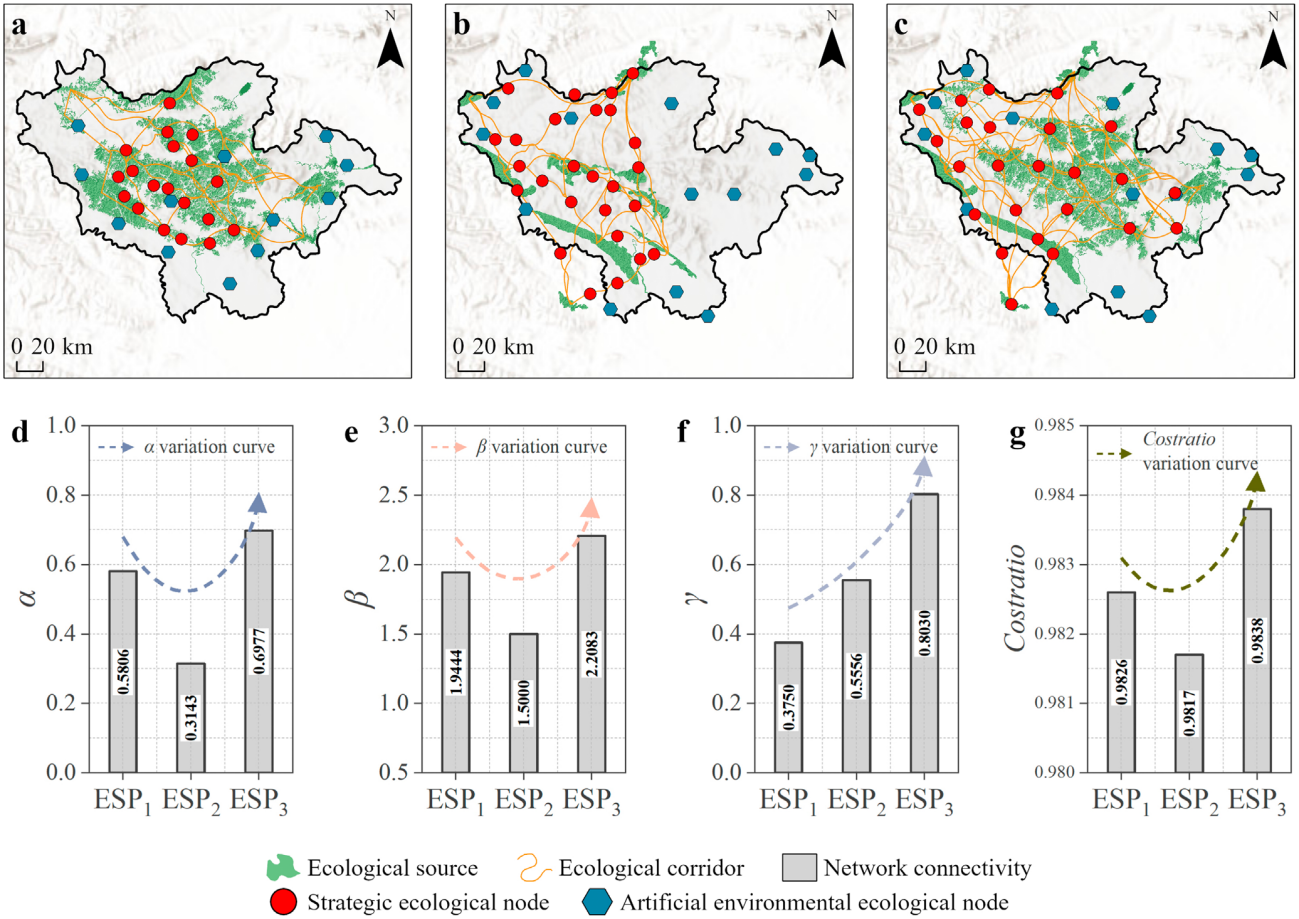
Spatial pattern and network connectivity of ESP (a–c, spatial distribution of ESP_1_, ESP_2_ and ESP_3_ in Funiu Mountain area; d–g comparison of *α*, *β*, *γ* and cost ratio in ESP_1_, ESP_2_, and ESP_3_).

There are significant variations of network configuration (including sources, corridors and nodes) in different ESPs (Figure 5). In ESP_1_, ecological sources were dispersed, ecological corridors initially formed a network structure, and strategic nodes coexisted with artificial environmental nodes, which basically realized the connectivity of ecological flows in Funiu Mountain area. In ESP_2_, ecological sources were fragmented, ecological corridors were fractured, and density of ecological nodes was sparse, which leads to a decrease in connectivity. In ESP_3_, aggregation of ecological sources was significantly improved, ecological corridors were intertwined to form a mesh structure, and strategic nodes and artificial environmental nodes were synergistically optimized, which constructed an ecological network with strong resilience.

Among the three patterns, ESP_3_ exhibited the highest *α* value (0.6977), followed by ESP_1_ (0.5806), whereas ESP_2_ showed the lowest value (0.3143). The *α* of ESP_3_ was 122% higher than that of ESP_2_, which showed redundant loop formation and improved ecological network resilience. For *β*, ESP_1_ (1.9444) > ESP_2_ (1.5000) < ESP_3_ (2.2083). The high *β* of ESP_3_ reflects the efficient connection between ecological corridors and ecological nodes, which can effectively decrease the resistance of ecological flow transmission. For the *γ* index, ESP_3_ showed the highest value (0.8030), followed by ESP_2_ (0.5556), while ESP_1_ exhibited the lowest value (0.3750). The *γ* of ESP_3_ was close to 0.8, indicating that the connectivity between ecological nodes has been improved, which was conducive to supporting the diffusion and gene exchange of species across ecological sources. For cost ratio, ESP_1_ (0.9826) > ESP_2_ (0.9817) < ESP_3_ (0.9838). Notably, network connectivity (*α*, *β* and *γ*) was significantly improved in ESP_3_ while its cost ratio only slightly increased by 0.21% compared to ESP_2_. Planning optimization measures can effectively balance ecological function enhancement and cost control.

## 4 Discussion

### 4.1 Vertical Differentiation Pattern of ESP and Its Causes

In Funiu Mountain area, the distribution of ecological sources shows significant vertical zonation characteristics, mainly concentrated at 800–1200 m. This altitude range has stable temperature and humidity conditions (mean annual temperature of 12.58°C and mean annual precipitation of 722.52 mm), which provide a suitable environment for the maintenance of mixed broadleaf and coniferous forest ecosystems (Ge et al. 2019). In contrast, low‐altitude areas (< 800 m) have flat terrain and high intensity of human activities (agricultural reclamation and urban construction). They may lead to large‐scale loss of natural habitats, high fragmentation of residual ecological patches, and ultimately make it difficult to form ecological sources that meet the scale and stability requirements (Liu et al. 2022). High‐altitude areas (> 1200 m) face harsh climatic challenges (e.g., low temperatures and strong winds) and generally infertile soil conditions (e.g., organic matter content often below 1.5%). They limit the development of vegetation and the ability to provide ecosystem services (Sun et al. 2025). As a result, the formation of large‐scale and highly connected ecological sources is equally hindered as in low‐altitude areas.

Ecological corridors were mainly distributed in low‐altitude and middle‐altitude areas (< 1200 m) and showed clear stepped terrain transition characteristics. These areas are rich in biological species and provide the necessary stepping stones and continuous paths for species migration (Imbach et al. 2013). The soil organic matter content in these areas is generally higher than in high‐altitude areas, which provides an excellent nutrient basis for maintaining the structural stability and ecological functions (e.g., filtering and transmission) of vegetation in ecological corridors (Choudhury et al. 2021). However, steep terrain, severe temperature differences and long snow coverage periods significantly increase migration resistance for large mammals in high‐altitude areas, leading to severely limited ecological connectivity functions (Storz et al. 2020). As a result, potential ecological corridors in high‐altitude areas showed island‐like distribution characteristics. The lack of effective, low‐resistance connections between ecological sources and their isolation from each other impedes ecological flow in high‐altitude areas (Zhang et al. 2021).

Most of the ecological nodes were located in areas below 1600 m. This spatial distribution highly aligns with the objective requirements of vertical zonation ecological protection in mountains (Elsen et al. 2018). Notably, all artificial environmental ecological nodes were in areas below 1200 m with intensive human activity in ESP_1_, ESP_2_ and ESP_3_. When organisms (especially ground‐dwelling species) spread or migrate from higher altitude ecological sources or ecological corridors to lowland plain areas with strong human activity interference, they face significant “ecological trap” risks (e.g., habitat loss and road barriers) (Zhang et al. 2013; Silva et al. 2014). The deployment of artificial environmental ecological nodes acts as key “stepping stones”, effectively bridging the gaps in ecological corridors caused by human activities, reducing migration resistance and enhancing the permeability and connectivity of lowland ecological networks (Zuo et al. 2022). Moreover, ecological nodes in three ESPs show a diverse distribution in spatial location, which helps to refine the configuration according to different conservation objectives and implementation conditions.

### 4.2 Mountain Vertical Zonation on ESP

Mountains are the most active vertical spectral units of ecological processes on the Earth's surface. The formation and evolution of their ESPs are driven by horizontal spatial heterogeneity and are more profoundly constrained by environmental stress on vertical gradient and vertical differentiation of human interference intensity (low altitude agriculturalization and mid‐high altitude natural conservation) (Buzhdygan et al. 2020; Sreekar et al. 2021). This composite pressure in vertical dimension makes ESPs of mountains show different characteristics from that of plain areas. In terms of ecological processes, biological exchanges between low‐altitude and high‐altitude areas rely on the connectivity of ecological corridors in middle‐altitude areas (Balangen et al. 2023; Céspedes Arias et al. 2022). However, many studies focused solely on a single horizontal dimension often overlook this hierarchical ecological flow characteristic (Han et al. 2024; Yu et al. 2022; Chen et al. 2021). In terms of conservation practices, ecological degradation in mountain areas often manifests as “obstructed downward migration” of species from middle‐altitude to high‐altitude areas due to low‐altitude human interference (Gao et al. 2009). Developing conservation strategies solely based on horizontal space might lead to an oversight of key vertical ecological nodes. In this study, artificial environmental ecological nodes below 1200 m provide supports for cross‐altitude migration, effectively compensating for this gap. Moreover, vertical differentiation of ESPs revealed in Funiu Mountain area confirms that the vertical gradient is the core driving axis of ESPs in mountain areas. Ignoring this dimension would reduce the construction of ESPs to a flat simulation, which struggles to address three‐dimensional complexity of mountain ecosystems.

At the theoretical level, the coupling analysis of vertical gradients and ecological security pattern confirms the universality of “middle‐altitude expansion effect” in the formation of ecological sources. This principle is verified not only in Funiu Mountain area but can also provide an important theoretical reference for pattern analysis of other global mountain ecosystems such as the eastern Himalayas and the Andes (Wang, Chang, et al. 2025; Wang, Liu, et al. 2025; Wang, Shen, et al. 2025). This study further found that ecological nodes in low‐altitude areas can effectively alleviate the interference pressure from human activities, thus becoming a key “ecological springboard” for high‐altitude species spreading to lower altitudes. Based on this, “vertical stepping stone effect” is proposed to explain the important role of low‐altitude ecological nodes in species spreading process. Although the case study of Funiu Mountain area has certain regional characteristics, the vertical differentiation rules of ESPs revealed by it still have important general reference value for other mountain areas facing similar vertical environmental gradients and human activity pressures worldwide. Against the backdrop of intensifying global climate change and the continuous expansion of human activities to mountain areas, a deep understanding and quantification of vertical gradient changes in ESPs has become an urgent practical need for assessing the vulnerability of mountain ecosystems and predicting their future evolution trends (Lu, Zhao, Chen, and Li 2025; Lu, Zhao, Chen, Li, Long, and Yang 2025).

### 4.3 Policy Recommendations

Based on vertical differentiation characteristics of ESPs and empirical findings in Funiu Mountain area, differentiated ecological functional zones were delineated (< 800 m as low mountain intervention zones, 800–1200 m as middle mountain core zones and > 1200 m as high mountain conservation zones) to clarify the dominant ecological functions and restoration priorities of each zone. The following policy recommendations are proposed:

For low mountain intervention zones, the expansion of new construction land should be strictly limited. Based on ecological resistances and species diffusion requirements, no less than 0.5 ha of “ecological stepping stone” construction land should be reserved along riverbank buffer zones, abandoned ditches, road intersections, both sides of river bridges and village edges. The design of “ecological stepping stone” should include native tree‐shrub‐grass multi‐layer vegetation (with a coverage > 80%), ponds and structures that avoid human interference. For middle mountain core zones, existing ecological sources and core ecological corridors should be strictly protected. Development activities that may cause fragmentation of ecological sources or disruption of ecological corridors should be prohibited. When constructing or renovating, departments like transportation and water resources should synchronously design and fund the construction of eco‐friendly crossing channels, vegetation restoration corridors or detour solutions around ecologically sensitive areas. For high mountain conservation zones, the focus should be on maintaining existing ecological baseline and reducing human interference, with an emphasis on preventing the impacts of climate change. In gentle areas with shorter snow accumulation periods, pilot projects of cold‐resistant and drought‐tolerant native shrubs planting and micro‐terrain modification should be carried out.

The middle‐altitude expansion effect and vertical stepping stone effect identified in this study should be understood as conceptual mechanisms that may also operate in other mountainous and topographically structured regions. In such contexts, intermediate elevation belts and transition zones often concentrate both ecological capacity and land‐use pressure, making them critical targets for spatial planning. European work that integrates ecosystem assessments and ecosystem service information into land use and urban planning provides relevant parallels, showing how ecological attributes can guide the designation of key ecological areas and corridors in relation to development patterns (Grêt‐Regamey et al. 2017). These experiences suggest that the middle‐altitude expansion effect and vertical stepping stone effect identified in the Funiu Mountain area may have broader applicability for refining ecological security patterns in municipal and regional planning, while also highlighting the need for further empirical validation in diverse socio‐ecological settings (Mak et al. 2021). In addition to biophysical constraints, variations in community‐based land management and local land use policies also influence ecological connectivity, as differences in forest stewardship, collective land decisions and protection enforcement can either enhance or weaken habitat continuity across altitudinal zones. Considering both ecological processes and social governance therefore provides a more comprehensive basis for understanding the spatial configuration of ecological sources and corridors in mountainous environments.

### 4.4 Limitations

This study still has some limitations. The division range of vertical gradient (200 m intervals) may mask the impacts of micro‐terrain on ESPs. The potential reshaping of ESPs under climate change due to the upward shift of vertical spectrum was not considered. The impacts of micro‐terrain on the distribution of ecological nodes can be focused on in future studies and combined with climate scenario models to simulate the dynamics of ESPs under future vertical spectrum migration (Lu, Zhao, Chen, and Li 2025; Lu, Zhao, Chen, Li, Long, and Yang 2025). Furthermore, a cross‐mountain comparison can be conducted to complete the general rules of ESPs in the vertical gradient of mountain areas. In practical applications, some ecological nodes or corridors may fall within lands with competing development claims or extend across administrative boundaries where planning objectives conflict, which can hinder the implementation and long‐term maintenance of ESPs.

In addition, although AHP weighting has been widely applied in resistance surface construction and has been validated in several ecological studies conducted within the Funiu Mountain area, we acknowledge that expert‐based weighting may still incorporate a degree of subjectivity. In future research, our team will further explore machine learning approaches to derive resistance factor weights based on empirical ecological data, which is expected to reduce uncertainty and enhance objectivity (Wang, Chang, et al. 2025; Wang, Liu, et al. 2025; Wang, Shen, et al. 2025). We also note that field‐based ecological monitoring, such as wildlife occurrence surveys and camera‐trap data, will be essential for validating the functional performance of ecological sources, corridors and nodes. These efforts will be incorporated into our subsequent research framework.

## 5 Conclusions

In this study, three types of ESPs were constructed, and their network connectivity was analyzed through the identification of ecological source areas in Funiu Mountain area. The differentiation characteristics of mountain ecosystems on vertical gradients were explained and targeted policy recommendations were subsequently proposed. In the ESP constructed by comprehensively considering connectivity and nature reserves, both the area of ecological sources and the length of ecological corridors show unimodal distributions with increasing altitude, peaking at 800–1000 and 1000–1200 m, respectively. The composite ecological network formed by this ESP conforms to the characteristics of mountain vertical zones. Moreover, its network connectivity performance is optimal (*α* = 0.6977, *β* = 2.2083, *γ* = 0.8030) and cost ratio (0.9838) only slightly increases compared to other types of ESPs, effectively achieving a balance between ecological function enhancement and cost control. In low‐altitude areas (< 800 m), it is important to strengthen the construction of ecological nodes to significantly enhance network connectivity. In middle‐altitude core area (800–1200 m), core area of ecological sources and key corridors should be strictly protected. In high‐altitude areas (> 1200 m), the stability of the ecosystem should be maintained, and the impacts of climate change should be actively addressed. The results aim to optimize the spatial configuration of mountain ecosystems, enhance their ecological service functions and adaptive capacity, providing a solid scientific basis for the protection and sustainable development of mountain ecology.

Overall, the integrated ecological security pattern developed in this study provides not only a framework for interpreting the spatial organization of mountain ecosystems in the Funiu Mountain area, but also a methodological basis that can be applied in other mountainous areas facing similar gradients of ecological sensitivity and human pressure. By combining connectivity analysis, ecological importance and niche suitability, this approach offers a practical tool for territorial spatial planning, ecological zoning and the protection of key ecological corridors. The refined understanding of vertical ecological structure, together with targeted management strategies for low‐altitude, middle‐ and high‐altitude zones, can support local governments in coordinating ecological protection and development needs, and in strengthening the resilience of mountain socioecological systems under ongoing environmental change.

## Author Contributions

**Zechen Wang:** writing – review and editing, writing – original draft, formal analysis, data curation. **Jingeng Huo:** writing – review and editing, writing – original draft, supervision, software, methodology. **Zhenqin Shi:** supervision, project administration, resources, funding acquisition. **Wenbo Zhu:** investigation. **Jiayuan Mao:** writing – review and editing, methodology, formal analysis. **Sicheng Yan:** data curation. **Shiliang Liu:** writing – review and editing, writing – original draft, formal analysis.

## Funding

This work was supported by the National Natural Science Foundation of China (NO. 42571351; U23A2020) and Natural Science Foundation of Henan Province, China (NO. 252300421463).

## Conflicts of Interest

The authors declare no conflicts of interest.

## Data Availability

I confirm that the Data Availability Statement is included in the main file of my submission, and that access to all necessary data files is provided to editors and reviewers.
